# The spiral symbiosis of skill and interest: the psychological mechanism of their synergistic development in PE classes

**DOI:** 10.3389/fpsyg.2026.1791070

**Published:** 2026-03-30

**Authors:** Yu Xue

**Affiliations:** School of Physical Education, Yan’an University, Yan’an, China

**Keywords:** autonomy support, perceived competence, physical education, self-determination theory, situational interest, skill acquisition, structural equation modeling

## Abstract

**Background:**

A longstanding contradiction has persisted in the field of physical education (PE). On the one hand, teaching oriented toward “fun PE” can overemphasize immediate enjoyment; yet because it often lacks substantive skill improvement, students’ interest becomes surface-level and difficult to sustain. On the other hand, traditional skill-centered teaching emphasizes technical proficiency, but its dull, repetitive process frequently extinguishes learners’ enthusiasm. This coexistence of two outcomes—students either have fun but do not really learn, or learn but do not enjoy it—has become a bottleneck hindering PE from developing toward higher quality.

**Purpose:**

To address this problem, the present study attempts to move beyond either-or thinking and clarify how skill and interest are connected. Based on foundational theories in contemporary motivational psychology—especially interpretations of the needs for competence and autonomy within Self-Determination Theory (SDT), and complementing this with other frameworks like Achievement Goal Theory—and combined with stage models of interest development, we propose and test a new theoretical framework: the Skill-Interest Spiral Symbiosis (SISS) Model. We aim to clarify how skill mastery is associated with learning interest through an indirect psychological pathway, and how interest in turn may feed back into further skill refinement, forming a potential feedback cycle.

**Methods:**

To verify the generalizability of the SISS Model, we conducted an anonymous cross-sectional survey among adult participants enrolled in sport-related courses using the Wenjuanxing platform. A total of 620 valid responses were collected (valid response rate: 88.6%), covering a wide range of sport programs such as basketball, yoga, swimming, and more. Key measures included: skill self-appraisal, perceived competence, perceived teacher autonomy support, situational interest, and long-term participation intention. Data were preliminarily processed with SPSS, and a structural equation model (SEM) was constructed in AMOS to empirically test the hypothesized paths. Bootstrap methods were further used to examine mediation and moderation effects.

**Results:**

Model fit indices for the SEM (*χ*^2^*/*df = 2.41, CFI = 0.95, RMSEA = 0.050), together with mediation and moderation tests, jointly validated a potentia feedback cycle of “skill → competence → interest → engagement.” The SEM showed that: (1) skill self-appraisal was an important positive predictor of perceived competence; (2) perceived competence played a key “bridge” role between skill self-appraisal and interest (partial mediation); (3) teacher autonomy support significantly “catalyzed” the conversion from competence to interest—under high autonomy-supportive environments, competence more readily was associated with interest; and (4) situational interest strongly and positively predicted behavioral engagement and long-term persistence, forming a complete pathway from psychological processes to behavior.

**Conclusion:**

The central argument is that skill and interest are not mutually exclusive choices; rather, they can form a symbiotic relationship that can mutually nourish and spiral upward. Skill improvement is the “fuel” that is associated with ignited interest, while interest is the “engine” that drives skill refinement. In this symbiotic cycle, perceived competence is the crucial psychological converter, and autonomy support in the teaching environment is the key “catalyst” determining conversion efficiency. While acknowledging the limitations of our cross-sectional data, establishing the SISS Model provides new ideas and practical leverage points for resolving the longstanding “learning vs. fun” dilemma in physical activity education, and offers guidance for designing more effective and attractive learning experiences.

## 1 Introduction

### 1.1 Research background and problem statement

Against the backdrop of the national fitness agenda rising to a state strategy, the importance of physical education is increasingly prominent. It not only concerns national physical health, but is also regarded as an important avenue for cultivating lifelong exercise habits and well-rounded personalities. However, the reality of PE is not optimistic. The continued decline in adolescents’ physical fitness has become a widely recognized public health concern, and “passive participation” and “lack of interest” in physical activity are also common among students (Diloy-Pena et al., 2024). Many students stop exercising after leaving school, indicating that school PE has not effectively transformed into lifelong personal habits. We believe a key reason is a long-standing classroom tension: teachers often feel they must choose between teaching skills well and making class interesting. This tension shapes many classroom choices.

**Figure 1:**
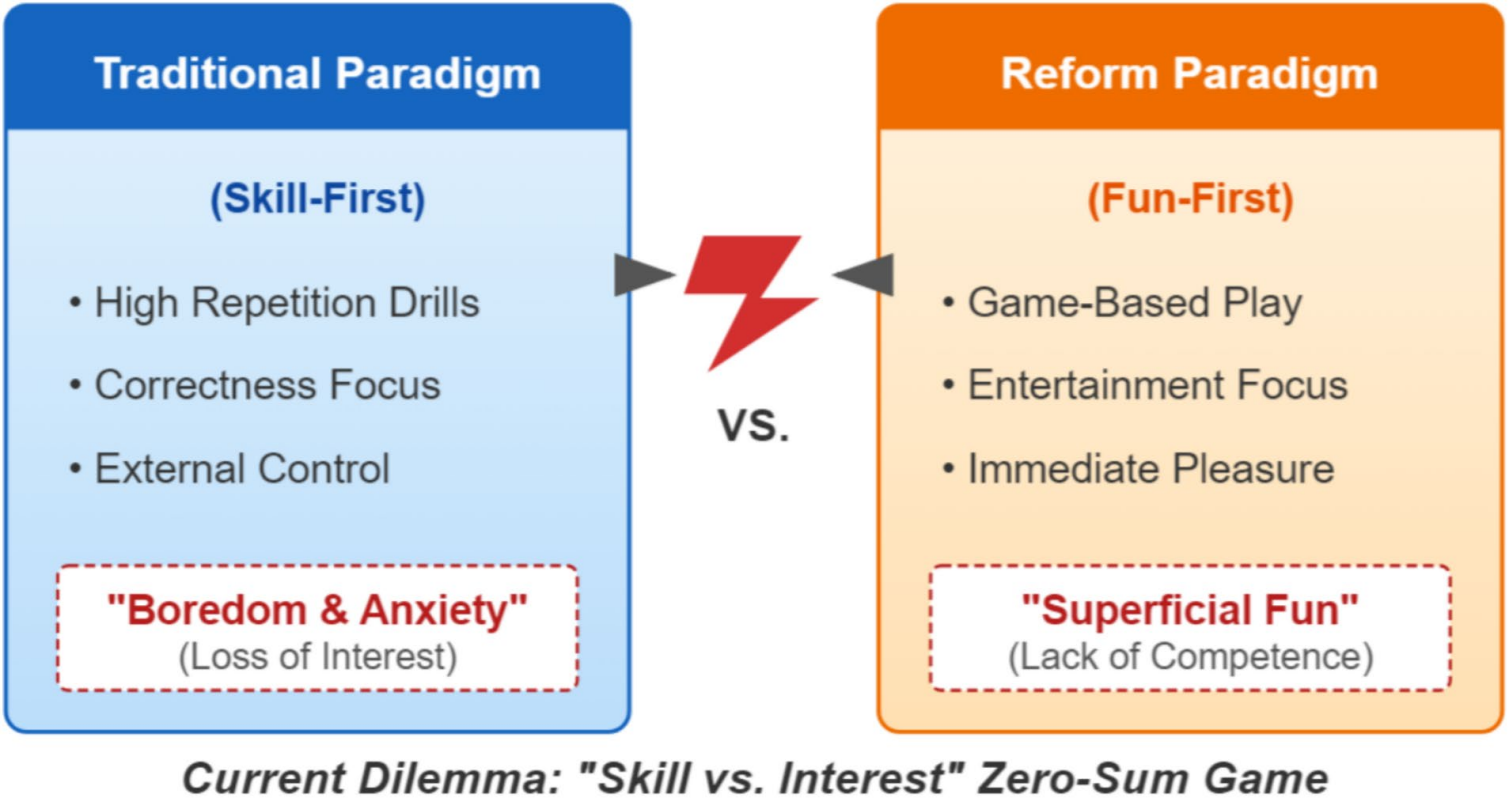
The binary dilemma of “skill–interest” in PE teaching.

This contradiction often makes PE practice swing between two extremes (as shown in Figure 1).

One extreme is the traditional “skill-first” teaching model, deeply influenced by competitive sports training systems, which focuses the core of instruction on the standardization, precision, and proficiency of techniques (Franco et al., 2025). Teachers attempt to “instill” standardized sport skills through large amounts of repetitive, segmented mechanical drills. This approach may improve test performance or technical level in the short term, but its highly controlling, low-enjoyment process is often accompanied by boredom, frustration, and anxiety (Cheon et al., 2022). Students may learn the movements through sweat, but may also “learn” to dislike the sport. Learning becomes a task that must be completed rather than an activity driven from within, which can severely suppress intrinsic motivation (Ryan et al., 2022; Ryan and Deci, 2000).

In response, another radical reform—“fun-first”—emerged. Under the banner of “happy PE,” it holds that interest comes first and teaching should be designed around fun and gamification, aiming to make students “enjoy playing” (Lin and Zhu, 2022). Classes feature novel games and entertaining activities; the atmosphere is lively and participation is high. The risk, however, is that overemphasis on immediate pleasure may neglect core skill instruction. Students may gain short-lived, surface-level enjoyment, but without systematic guidance and challenging progressive practice, they are unlikely to experience deeper accomplishment arising from improved competence (Strotmeyer et al., 2022). Such “shallow interest without skills” is rootless and easily dissipates when the context changes, failing to become stable individual interest that drives learners to practice independently after class, overcome difficulties, and pursue mastery (Roure and Pasco, 2022).

So teachers can feel stuck: focus on skill and lose motivation; focus on fun and lose learning outcomes. We think the real issue is not skill or interest themselves, but how we understand their relationship. Could there be a mechanism where skill and interest actually support each other? That is the question this study addresses.

### 1.2 Moving past “either skill or fun”

To move forward, we need to look at basic motivation psychology and rethink how skill and interest connect.

We argue that teaching skills well is not the enemy of interest. In many cases, it is what creates deeper interest. When learners put in effort and clearly see progress, they get a strong sense of “I can do this.” That feeling is different from the quick excitement of a game; it is more closely tied to personal growth (Bourke et al., 2025). So even drills can feel rewarding if students can see improvement. Without skill progress, interest that relies only on novelty is often short-lived.

At the same time, real interest is not just “being entertained.” Interest can become an internal push that helps people keep practicing and improving. Interest may start as a reaction to something new (Chen et al., 1999), but to last, it needs repeated success and some room for choice and exploration. When learners often feel successful and feel some control over how they practice, they invest more time and effort, and they start to actively pursue improvement (Kruse et al., 2024; Guan et al., 2023).

## 2 Theoretical foundations and hypothesis development

To connect skill and interest, we use Self-Determination Theory (SDT), which says people have basic psychological needs like feeling capable, having choice, and feeling connected to others (Ryan et al., 2022; Williams and Deci, 1996).

Here, feeling capable is the key link between skill and interest. When PE teaching helps students learn skills, students are more likely to feel “I’m good at this,” which can make the activity feel more interesting (Lourenço et al., 2022; Menescardi et al., 2023).

Having choice/support affects how easily capability turns into interest. If teachers provide choices, encourage questions, and explain why drills matter, students are more likely to feel “my progress comes from my own effort,” which helps interest grow (Mossman et al., 2024; Patzak and Zhang, 2025). If teaching is overly controlling, students may still perform the movement but feel less internal satisfaction, so interest grows more slowly.

**Figure 2:**
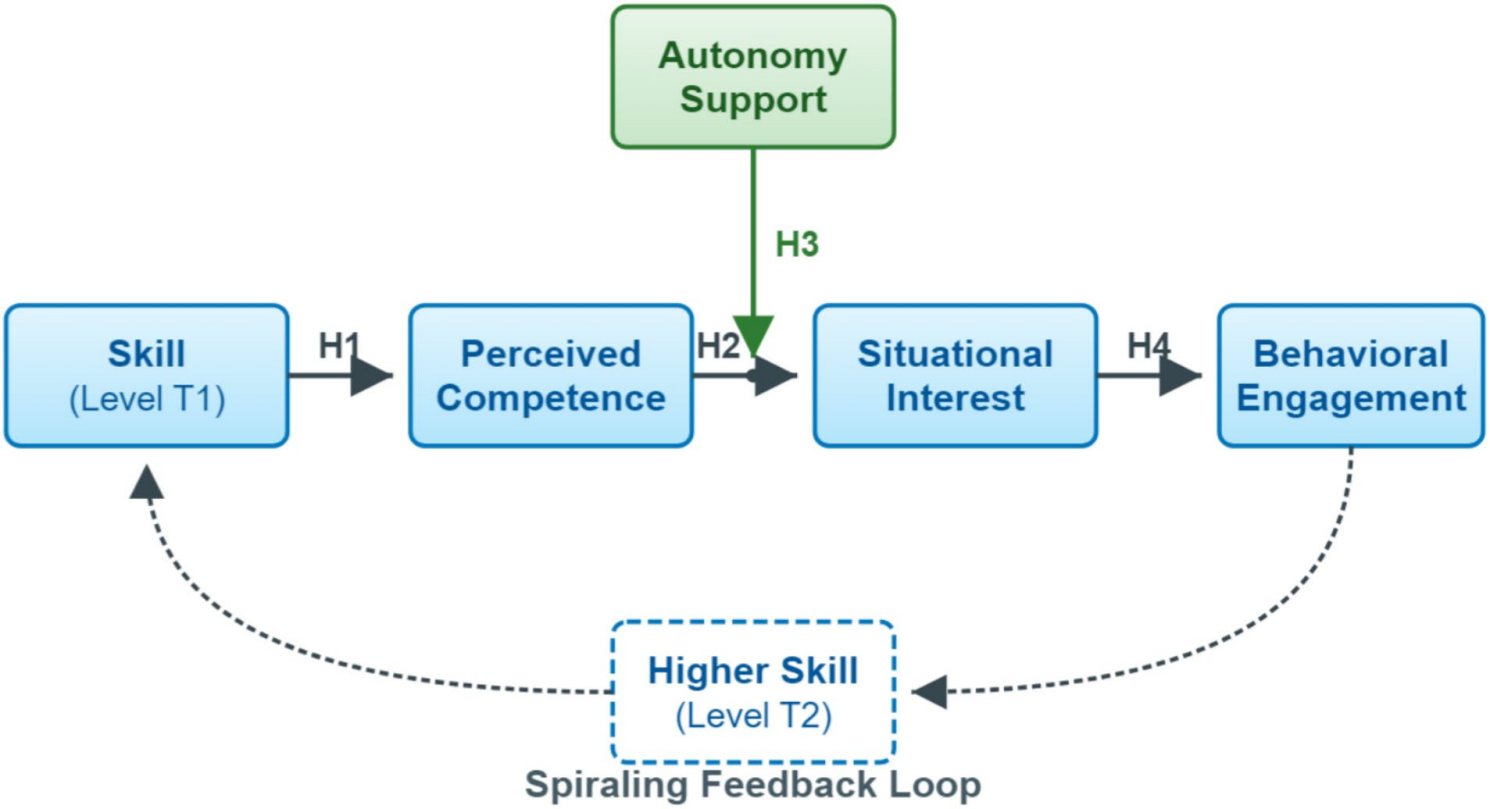
The theoretical framework of the Skill–Interest Spiral Model. The arrow denotes a hypothesized process of reciprocal influence over time, which this cross-sectional study aims to test as a static covariance structure.

This leads to our model—the Skill–Interest Spiral Model (Figure 2). It describes a positive feedback cycle. We propose:

*H1 (Skill → Competence)*: Individuals’ level of skill self-appraisal mastery positively predicts perceived competence. Put simply, the better individuals perceive their own skill, the more individuals feel “I can do it” (Bourke et al., 2025).

*H2 (Competence → Interest)*: Perceived competence positively predicts situational interest in sports activities. That is, the more individuals feel “I can do it,” the more they find the activity engaging (Kruse et al., 2024; McAuley et al., 1989).

*H3 (Moderating role of autonomy support)*: Teacher autonomy support positively moderates the relationship between perceived competence and situational interest. Specifically, the stronger the autonomy support, the more efficiently competence converts into interest (Cheon et al., 2022).

*H4 (Interest → Engagement)*: Situational interest positively predicts behavioral engagement and long-term participation intention. The more “interesting” individuals find it, the more willing they are to invest in practice and plan long-term participation—potentially initiating the next round of higher-level skill learning (S+) (Guan et al., 2023; Standage et al., 2003).

## 3 Methods

### 3.1 Participants

We used convenience sampling. Surveys were sent to university students and adults taking sports courses at fitness clubs. Participation was anonymous and voluntary with electronic consent. Out of 700 responses, we screened out low-quality responses and kept 620 valid questionnaires (88.6%).

As shown in Table 1, among the 620 participants, 339 were male (54.7%) and 281 were female (45.3%). Ages ranged from 18 to 48, with a mean age of 21.42 (SD = 2.09), indicating a predominantly young sample. Sports programs were diverse, including basketball, badminton, yoga, swimming, fitness training, and more.

**Table 1:** Participant demographics (*N* = 620).

Variable	Category/statistic	Frequency (*n*)	Percentage (%)/value
Gender	Male	339	54.7%
	Female	281	45.3%
Age	Mean (*M*)	–	21.42
	Standard deviation (SD)	–	2.09
	Minimum	–	18
	Maximum	–	48
Years of practice	<1 month	98	15.8%
	1–6 months	155	25.0%
	6 months–1 year	162	26.1%
	1–3 years	143	23.1%
	>3 years	62	10.0%

### 3.2 Measures

All measures used in this study were mature scales widely applied in psychology and PE, with well-established reliability and validity. The original English scales were translated into Chinese and then back-translated by two bilingual experts to ensure accuracy and cultural appropriateness. All items used a 7-point Likert scale (1 = strongly disagree, 7 = strongly agree). The complete questionnaire used in this study is available in the Supplementary material.

Skill Self-Appraisal: Three items were self-developed based on PE teaching practice to assess participants’ subjective evaluation of their technical mastery and application in a given sport, serving as a proxy indicator for objective skill level. Items focused on the standardization of basic movements, technical fluency, and practical application ability. Example: “Do you think your basic movements (e.g., posture, footwork) are standard and correct?” We acknowledge that this measures self-perception of skill rather than objective performance, a point further addressed in the limitations section.

Perceived Competence: Measured using five items from the competence subscale of the Intrinsic Motivation Inventory (IMI) developed by McAuley et al. (1989). Example: “I think I am good at this sport.” Conceptually, while this construct overlaps with skill self-appraisal, the latter focuses on the technical aspects of performance, whereas perceived competence captures a more global feeling of capability and effectiveness in the activity.

Perceived Autonomy Support: Measured using an adapted version of the Learning Climate Questionnaire (LCQ). The original scale was developed by Williams and Deci (1996); this study adopted the version used in PE contexts by Standage et al. (2006). Example: “The teacher/coach gave me opportunities to choose how to do things.”

Situational Interest (Situational Interest): Based on Chen et al.’s (1999) Situational Interest Scale (SIS), selecting items focusing on novelty and attention demand. Example: “The content of this class made me feel excited and interested.”

Behavioral Engagement and Intention: This construct included two parts. Behavioral engagement was adapted from Skinner et al. (2009); future participation intention was adapted from Ntoumanis (2001). Examples: “When I encounter a difficult movement, I keep trying until I do it correctly” (engagement), and “I plan to continue practicing this sport in the coming period” (intention).

### 3.3 Procedure

The questionnaire was distributed online via the Wenjuanxing platform. Instructions asked participants to recall the most memorable PE class or training experience in the recent past (within one week) and respond based on that experience. To minimize common method bias, we implemented several procedural controls: participants were assured of anonymity and confidentiality, the order of the measurement items was randomized for each participant, and the survey instructions emphasized that there were no right or wrong answers. Data collection was completed in October 2025.

### 3.4 Data analysis

Analyses were conducted using SPSS 26.0 and AMOS 24.0. The raw dataset supporting the conclusions of this article is available in the Supplementary material.

Preliminary analysis: Descriptive statistics, normality tests, and Pearson correlations among variables (SPSS).

Reliability and validity: Confirmatory factor analysis (CFA) to evaluate structural validity, composite reliability (CR), and average variance extracted (AVE) (AMOS).

Model testing: Structural equation modeling (SEM) in AMOS to test overall model fit and the significance of key path coefficients.

Advanced tests: Bias-corrected percentile Bootstrap (5,000 resamples) to test mediation of perceived competence; a separate hierarchical multiple regression analysis was conducted in SPSS to test the moderation effect of autonomy support (H3). Variables were mean-centered before creating the interaction term (Competence × Autonomy Support) to reduce multicollinearity.

## 4 Results

### 4.1 Preliminary analysis

Before formally testing the model, we examined data characteristics, normality, reliability/validity, and correlations. As shown in Table 2, the absolute values of skewness and kurtosis for all core variables were far below recommended thresholds, indicating good univariate normality and supporting the use of maximum likelihood estimation in subsequent modeling.

**Table 2:** Descriptive statistics and normality tests (*N* = 620).

Variable	Mean (*M*)	SD	Skewness	Kurtosis
Skill self-appraisal (Skill)	4.62	1.15	−0.12	−0.45
Perceived competence (Comp)	4.45	1.08	−0.09	−0.38
Teacher autonomy support (Auto)	4.81	1.12	−0.21	−0.52
Situational interest (Inter)	4.73	1.05	−0.15	−0.41
Behavioral engagement (Engage)	4.92	1.01	−0.25	−0.33

Reliability and validity were also ideal (Table 3). Cronbach’s *α* and composite reliability (CR) values for latent variables were all above 0.70, indicating good internal consistency. AVE values exceeded 0.50, and the square roots of AVE were larger than inter-construct correlations, supporting convergent and discriminant validity.

**Table 3:** Reliability and validity of latent variables.

Latent variable	Cronbach’s *α*	CR	AVE
Skill self-appraisal (Skill)	0.78	0.81	0.59
Perceived competence (Comp)	0.83	0.84	0.51
Teacher autonomy support (Auto)	0.79	0.80	0.53
Situational interest (Inter)	0.86	0.87	0.57
Behavioral engagement (Engage)	0.85	0.86	0.55

The correlation matrix (Table 4) provided preliminary support for our hypotheses. All core variables showed moderate to strong significant positive correlations, consistent with theoretical expectations. For example, skill self-appraisal correlated most strongly with perceived competence (*r* = 0.68), while situational interest correlated most strongly with behavioral engagement (*r* = 0.75).

**Table 4:** Pearson correlation matrix.

Variable	1. Skill	2. Comp	3. Auto	4. Inter	5. Engage
1. Skill	1				
2. Comp	0.68^**^	1			
3. Auto	0.35^**^	0.42^**^	1		
4. Inter	0.55^**^	0.62^**^	0.48^**^	1	
5. Engage	0.49^**^	0.58^**^	0.41^**^	0.75^**^	1

^**^Indicates *p* < 0.01.

### 4.2 Structural model testing

**Figure 3:**
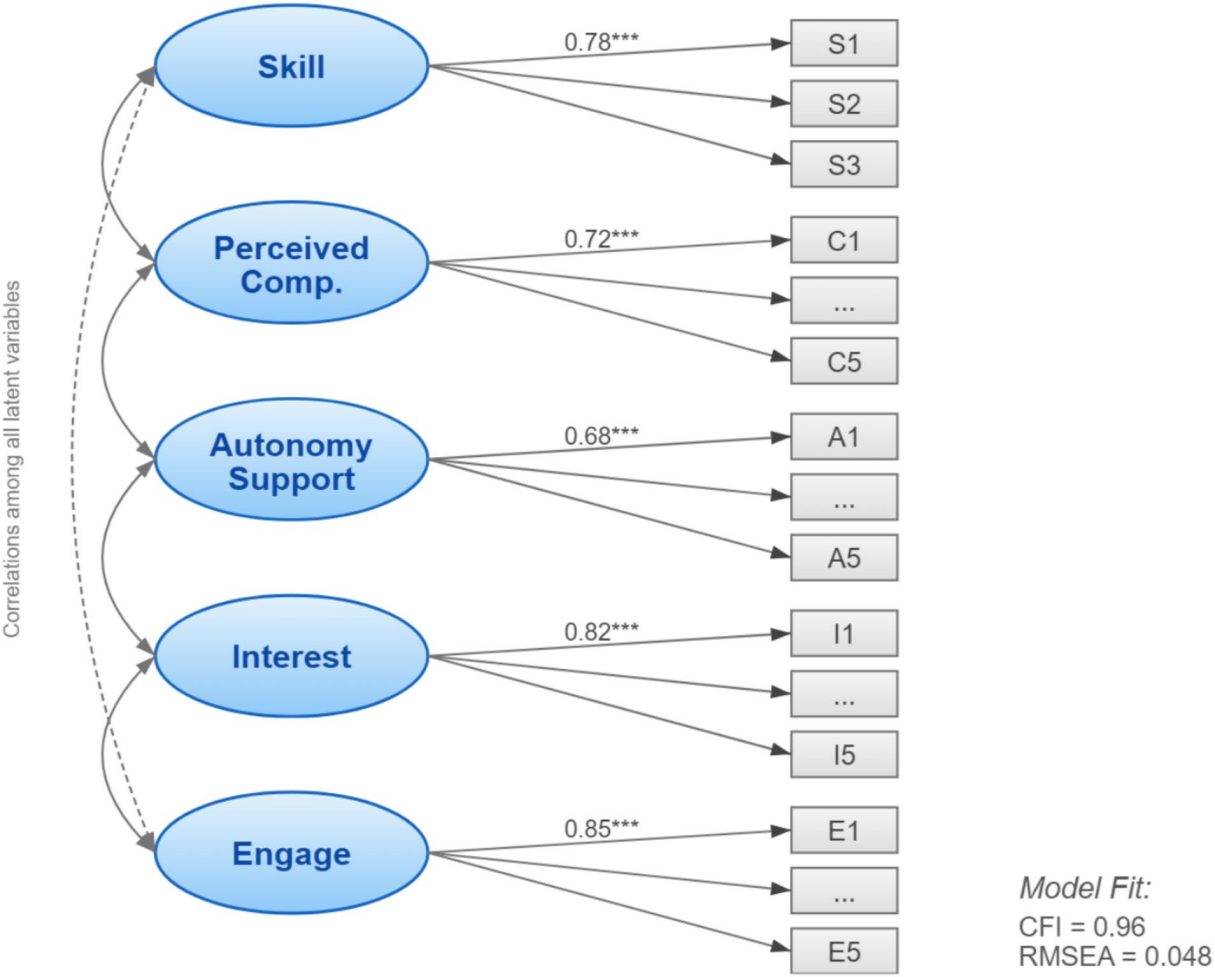
Measurement model (CFA) structure diagram.

After confirming good reliability and validity of the measurement model (structure shown in Figure 3; fit indices in Table 5), we tested the core SISS Model.

**Table 5:** Fit indices for measurement and structural models.

Model	*χ*^2^/df	CFI	TLI	RMSEA	SRMR
Measurement model (CFA)	2.35	0.96	0.95	0.048	0.042
Structural model (SEM)	2.41	0.95	0.94	0.050	0.045
Recommended criteria	<3	>0.9	>0.9	<0.08	<0.08

The structural model showed satisfactory fit. Key indices (*χ*^2^*/*df = 2.41, CFI = 0.95, TLI = 0.94, RMSEA = 0.050, SRMR = 0.045) met or exceeded recommended standards, indicating that the theoretical model fit the observed data well.

**Figure 4:**
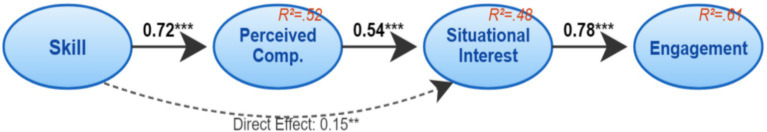
Path analysis results of the SISS model. All path coefficients are standardized and significant. The model explained 52% of the variance in perceived competence (*R*^2^ = 0.52), 48% in situational interest (*R*^2^ = 0.48), and 61% in behavioral engagement (*R*^2^ = 0.61). ^***^p < 0.001.

Key path results (Figure 4 and Table 6) strongly supported our hypotheses:

**Table 6:** Structural path coefficients.

Hypothesis	Path	Standardized coefficient (*β*)	S.E.	*t* (C.R.)	*p*	Conclusion
H1	Skill → Perceived competence	0.72	0.04	18.45	^***^	Supported
H2	Perceived competence → Situational interest	0.54	0.05	12.33	^***^	Supported
H3	Competence × Autonomy Support → Interest	0.14	0.03	4.52	^***^	Supported (see Sec 4.4)
H4	Situational interest → Behavioral engagement	0.78	0.04	21.05	^***^	Supported
–	Teacher autonomy support → Situational interest	0.28	0.04	6.89	^***^	Significant

^***^Indicates *p* < 0.001. H3 was tested via regression analysis (see Section 4.4).

*H1 supported*: Skill self-appraisal strongly and positively predicted perceived competence (*β* = 0.72, *p* < 0.001).

*H2 supported*: Perceived competence significantly and positively predicted situational interest (*β* = 0.54, *p* < 0.001).

*H3 supported*: Situational interest strongly predicted behavioral engagement (*β* = 0.78, *p* < 0.001).

Together, the predictors explained 52% of the variance in perceived competence, 48% in situational interest, and 61% in behavioral engagement, indicating strong explanatory power.

### 4.3 Mediation analysis

**Figure 5:**
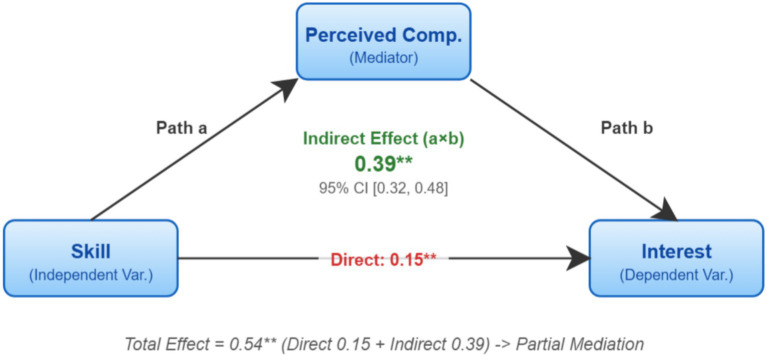
Mediation pathway of perceived competence.

We next tested whether perceived competence played the expected “bridge” role between skill and interest. Using bias-corrected percentile Bootstrap (5,000 resamples), we tested mediation; results are shown in Figure 5 and Table 7.

**Table 7:** Bootstrap test of mediation (*N* = 620).

Effect pathway	Effect type	Standardized effect	95% CI	Conclusion
Skill → Competence → Interest	Indirect effect	0.39	[0.32, 0.48]	Significant
Skill → Interest	Direct effect	0.15	[0.08, 0.23]	Significant
Skill → Interest	Total effect	0.54	[0.47, 0.61]	Significant

The indirect effect of skill on situational interest via perceived competence was significant (0.39), with a 95% confidence interval of [0.32, 0.48] not including 0. Meanwhile, the direct effect was also significant (0.15) but substantially smaller than the indirect effect. This clearly confirms that perceived competence served as a key partial mediator between skill and interest.

### 4.4 Moderation analysis (H3 test)

Finally, we tested a key hypothesis: whether teacher autonomy support “catalyzes” the conversion from competence to interest. As described in the analysis section, we conducted a hierarchical multiple regression analysis. In the final step, the interaction term (Competence × Autonomy Support) was entered. The results showed that the interaction term was a significant predictor of situational interest (*β* = 0.14, *t* = 4.52, *p* < 0.001, Δ*R*^2^ = 0.018), indicating a moderation effect and supporting H3.

**Figure 6:**
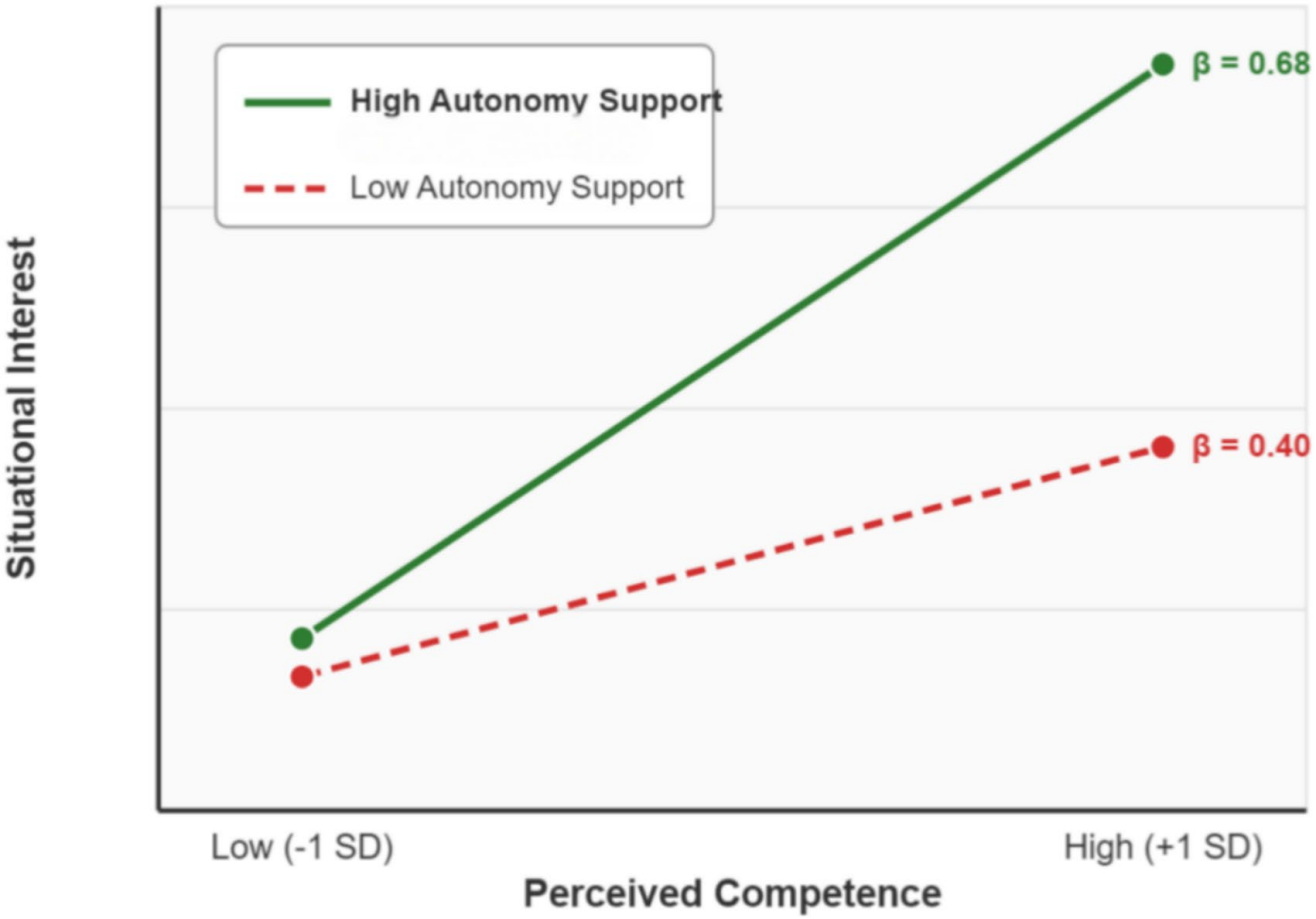
Moderating effect of teacher autonomy support on the competence–interest relationship. The relationship between perceived competence and situational interest was significant and positive under both high (+1 SD) autonomy support (*β* = 0.68, *p* < 0.001) and low (−1 SD) autonomy support (*β* = 0.40, *p* < 0.001).

To probe the nature of this significant interaction, we performed a simple slope analysis. The results are plotted in Figure 6.

As the plot clearly illustrates, the positive effect of perceived competence on situational interest is substantially stronger when students perceive high levels of teacher autonomy support compared to when they perceive low support. This confirms that teacher autonomy support acts as a crucial catalyst, amplifying the positive impact of perceived competence. Therefore, H3 was fully supported.

## 5 Discussion

Based on an empirical survey of 620 adult sport participants, this study successfully constructed and validated the Skill–Interest Spiral Symbiosis Model. The findings not only reveal the internal linkage among skill, competence, interest, and engagement at the data level, but more importantly provide an integrative new theoretical perspective—beyond either-or thinking—for understanding and resolving the longstanding “learning vs. fun” contradiction in physical activity education.

### 5.1 The symbiotic mechanism between skill and interest: the core of “discrimination”

A persistent misconception is that teaching practice must choose between skill instruction and interest cultivation. Our results clearly show that the two are not antagonistic but internally connected.

Our data demonstrate a strong psychological pathway: Skill self-appraisal → Perceived competence → Situational interest. Subjective Skill improvement (*β* = 0.72) is the most direct and effective source associated with igniting the “I can do it” flame of competence, echoing Bourke et al.’s (2025) meta-analytic findings. This inner competence, in turn, is a powerful driver of interest (*β* = 0.54), aligning with longitudinal evidence from Kruse et al. (2024). Mediation analysis further shows that most of skill self-appraisal’s association with interest is realized through the psychological converter of perceived competence (indirect 0.39 vs. direct 0.15). In other words, the most reliable way to make students feel “this is interesting” is to let them experience “I can do it.” While we acknowledge the conceptual proximity of self-appraised skill and perceived competence, our model suggests a sequential relationship where specific technical self-evaluations feed into a more global sense of capability, which then fosters interest. A well-designed skill-learning process that allows frequent small, clear improvements experiences carries intrinsic joy. Unlike fleeting excitement from pure play, this joy is deeper and more durable, tied to self-growth and competence confirmation. In this sense, effective practice is joyful in itself—a claim strongly supported here. Teachers should not avoid skill teaching; rather, they should make it *learnable* and *perceivable*, continuously supplying high-quality “fuel” for interest.

If skill self-appraisal supplies interest through competence, then interest in turn provides the driving force for sustained skill refinement. Situational interest had an extremely strong predictive effect on behavioral engagement and long-term intention (*β* = 0.78), the strongest effect in the entire model. Once individuals become genuinely interested, they spontaneously invest more cognitive resources to improve movements, exert greater effort to overcome difficulties, and plan to continue participation. This “I want to learn” state is far more efficient and persistent than “I have to learn” driven by external pressure, explaining why interest is a gateway to deliberate practice and lifelong sport participation (Guan et al., 2023). Interventions grounded in SDT also show that motivation activated by satisfying basic psychological needs effectively enhances long-term sport participation intention (Fernández-Espínola et al., 2022). Without interest, skill learning is like rowing upstream; with interest as a powerful engine, skill learning enters a self-accelerating, self-reinforcing fast lane.

### 5.2 The teacher’s key role: the “catalytic” function of autonomy support

If competence is the “bridge” linking skill and interest, the moderation results reveal the decisive role of teaching style. Teacher autonomy support significantly promotes the conversion from competence to interest, functioning like a “catalyst” (Mossman et al., 2024).

This means that for two students with identical skill levels and competence experiences, whether they ultimately develop strong interest in the sport depends largely on their teaching environment (Franco et al., 2025). In autonomy-supportive settings, teachers explain practice purposes, offer choices, encourage questions, and affirm effort and progress. Students then attribute success to internal effort and ability, maximizing the internalization from “I did it” into “I like it.” Here the teacher acts as an enabler and guide (Yang et al., 2022).

In low autonomy support (i.e., controlling) environments, teachers may rely more on instructions and commands. Even if students complete movements, they may feel like passive just doing what they are told; success experiences are diluted by external control, making it harder to convert into genuine intrinsic pleasure and interest, and may even lead to behavioral problems (Curran et al., 2013). In such contexts, teachers inadvertently become dictators and monitors, objectively obstructing the satisfaction of psychological needs and forming a “barrier” to competence-to-interest conversion.

Therefore, the findings provide a profound implication for teacher role positioning: teachers are not decision-makers choosing between “skill” and “interest,” but environmental engineers and psychological catalysts who promote their spiral coevolution (Patzak and Zhang, 2025; Guo et al., 2023). Their core task is to ensure students achieve skill success through scientific instructional design (satisfying competence) and to infuse that success experience with meaning and enjoyment through autonomy-supportive teaching (satisfying autonomy), thereby igniting and sustaining the spiral symbiosis system.

### 5.3 The upward spiral logic: the S–C–I–E loop

Synthesizing all findings, we can construct a theoretical dynamic, self-reinforcing Skill–Interest coevolution spiral loop (S–C–I–E Loop):

Skill learning (S) → Perceived competence (C) → Situational interest (I) → Behavioral engagement (E) → Higher-level skill (S+).

The logic is: effective teaching enables students to master skills (S); objective capability gains convert into perceived competence (“I can do it”) (C); in an autonomy-supportive atmosphere, competence is efficiently associated with situational interest (“this is interesting”) (I); strong interest then triggers higher engagement (E), with more time spent practicing and exploring; high engagement is expected to lead to further skill improvement (S+). Higher skills (S+) would then yield stronger competence (C+), which would elicit deeper and more stable interest (I+), driving even greater engagement (E+), ultimately moving toward expert-level skill and enduring individual interest. Recent longitudinal studies have begun to reveal such reciprocal, time-developing relationships between actual and perceived motor competence (Strotmeyer et al., 2022).

The model’s core contribution is that it transforms skill and interest from static, opposing elements into a dynamic, mutually generating process. Our cross-sectional data provide a snapshot that supports the plausibility of this structure, showing that the most efficient skill learning may occur precisely when interest is strongest, and the most enduring interest appears to be rooted in continual breakthroughs in skill. The ultimate goal of physical activity education is to initiate and maintain this positive spiral, enabling students—driven by intertwined accomplishment from “learning it” and joy from “enjoying it”—to keep climbing new peaks of skill and interest.

## 6 Practical implications and limitations

### 6.1 Teaching implications

How can the SISS Model be implemented in frontline teaching? Our study points to three clear and actionable pathways that help teachers shift from “choosers” to “facilitators”:

Course design: Embrace stepwise instruction of “micro-skills–micro-success.” Teachers should break complex sport skills into a sequence of small units with incremental difficulty and clear logic. The key is ensuring that most students experience at least one “I learned it” moment in each lesson. High-frequency competence experiences are the fundamental basis for continuously “powering” interest.

Teaching style: Shift from “directive” to “autonomy-supportive.” Incorporate autonomy-support elements: offer 2–3 practice options, (e.g., “For the next 10 min, you can choose to work on your serves or practice your volleys”), explain “why” not only “how,” (e.g., “We are doing this footwork drill because it is the foundation for moving quickly to the ball”), encourage questions, and shift attention from mistakes to effort and progress.

Assessment: Shift from “horizontal comparison” to “vertical comparison.” Use more formative and self-referenced evaluation so assessment becomes a “mirror” for perceiving self-growth rather than a “ruler” for comparing with others—for example, using video records so students can directly see their improvements, thereby protecting and stimulating competence.

### 6.2 Limitations and future directions

No study is perfect. We acknowledge the following limitations and suggest corresponding directions for future research. This section is structured to clearly summarize the study’s boundaries and potential avenues for advancement.

Conceptual and methodological limitations of the cross-sectional design: The primary limitation is the cross-sectional nature of our data. While our SEM tests the covariance structure proposed by the SISS model, it cannot establish causality or capture the dynamic, temporal nature of a “spiral.”Although we verified that interest predicts engagement, a bidirectional relationship is also plausible. Future research should adopt longitudinal tracking or cross-lagged panel designs (as in Kruse et al., 2024; Strotmeyer et al., 2022) to more definitively reveal the reciprocal and time-based causal dynamics of the skill-interest relationship.

Measurement validity and common method bias: The study relied exclusively on self-report measures. This introduces two issues. First, there is a risk of common method bias (CMB). Although we applied procedural controls (anonymity, randomized item order) and Harman’s single-factor test suggested bias was not severe, the perceptual nature of the data may inflate relationships. Second, the use of “skill self-appraisal” as a proxy for objective skill creates a conceptual overlap with “perceived competence,” risking a tautological relationship. Future studies should incorporate multi-method and multi-source data to strengthen objectivity and validity. This could include objective skill assessments (e.g., time trials, accuracy scores), expert or teacher ratings of student skill, and observational measures of engagement.

Generalizability of the sample: Our sample consisted of university students and adults in fitness clubs (mean age 21.42), which is not representative of the K-12 school PE population. Therefore, conclusions should be applied cautiously to younger learners. Future research is needed to validate the SISS model across different populations, especially children and adolescents at various developmental stages, as the factors that initiate and sustaining the spiral may differ by age (den Uil et al., 2023; Wang et al., 2023).

Deepening and extending the theoretical model: This study focused primarily on the needs for competence and autonomy. Future research could extend the model by incorporating the need for relatedness (e.g., team climate, peer support) to examine how the full triad of basic psychological needs jointly shapes the skill–interest symbiosis (Gu et al., 2023; Bento et al., 2024). Furthermore, testing specific instructional interventions known to satisfy basic needs (e.g., the Sport Education Model; Manninen and Campbell, 2022) would be a valuable next step to evaluate how effectively they activate the S-C-I-E spiral in practice. Finally, comparing the model’s parameters across different sport types (e.g., individual vs. team sports) could reveal context-specific nuances (Gu et al., 2023).

## 7 Conclusion

The core contribution of this study is that, supported by empirical evidence, it breaks the binary-opposition myth of “skill” versus “interest” in physical activity education and successfully constructs the Skill–Interest Spiral Symbiosis Model (SISS Model) to explain their potential coevolution. Our conclusions can be summarized as follows:

Skill self-appraisal and interest are not opposites; through an indirect psychological pathway, they appear to mutually promote each other and spiral upward.

Perceived competence is the key psychological bridge between skill mastery and situational interest, showing significant partial mediation.

Teacher autonomy support is an important external catalyst in this symbiotic process, significantly strengthening the association between competence to interest.

The resulting theoretical feedback cycle—Skill (S) → Competence (C) → Interest (I) → Engagement (E) → Higher skill (S+)—provides an integrative framework that transcends traditional dilemmas.

Ultimately, the essence of effective physical activity education may not lie in difficult trade-offs between “skill” and “interest,” but in how scientific, wise instruction precisely initiates and accelerates this internal psychological spiral. When a student can experience both the invigorating achievement of skill breakthroughs and the joy of exploration and expression in an autonomy-supportive, respectful atmosphere, we truly realize the highest goal of such education: not only teaching them “how to move,” but igniting an enduring passion for movement—paving an ideal path from “learning it,” to “loving it,” to “lifelong companionship.”

## Data Availability

The original contributions presented in the study are included in the article/Supplementary material, further inquiries can be directed to the corresponding author.
