# Supplementary material for: The spiral symbiosis of skill and interest: the psychological mechanism of their synergistic development in PE classes
Source: Front Psychol. 2026 Mar 30;17:1791070. doi: 10.3389/fpsyg.2026.1791070 (PMC13070935; doi:10.3389/fpsyg.2026.1791070)
Supplement: Supplementary file 1 [file Data_Sheet_1.zip › Supplementary_Material_1_Questionnaire.docx.docx]

# Physical Education Course Learning Experience Survey Questionnaire

## **[Questionnaire Instructions]**

This questionnaire aims to understand your recent learning experiences and psychological feelings in physical education classes/training. As an academic study, your responses are of great value for optimizing the design of physical education instruction. This survey is completely anonymous. All data will be used only for aggregated statistical analysis. Please answer truthfully and with confidence.

## **Part 1: Informed Consent**

1. I am at least 18 years old.
2. I understand the purpose of this study. I know that participation is completely voluntary, and I may withdraw at any time.
3. I agree to participate in this study and provide my feedback.

**□ I agree and begin answering** (If you do not agree, please close the page.)

## **Part 2: Basic Information**

1. **Gender:** □ Male □ Female □ Other
2. **Age:** _____ years old
3. **Your current status:** □ Undergraduate/Graduate student □ Employed □ Self-employed/Other
4. **Sport/activity for this survey:** _____ (e.g., basketball, yoga, badminton, swimming, etc.)
5. **How long have you practiced this activity?**
   □ Less than 1 month

□ 1–6 months

□ 6 months–1 year

□ 1–3 years

□ More than 3 years

## **Part 3: Core Measurement Scales**

**Instructions:** Please recall the PE class/training session you just completed, or the most impressive practice experience you had recently (within one week). Based on your **true feelings**, rate the following statements.
**(Rating scale: 1 = Strongly disagree, 2 = Disagree, 3 = Somewhat disagree, 4 = Neutral, 5 = Somewhat agree, 6 = Agree, 7 = Strongly agree)**

### **A. Self-Appraisal of Objective Skill Level**

| Item | 1 | 2 | 3 | 4 | 5 | 6 | 7 |
| --- | --- | --- | --- | --- | --- | --- | --- |
| 1. Do you think your basic movements (e.g., posture, footwork, etc.) are standard and correct? | ○ | ○ | ○ | ○ | ○ | ○ | ○ |
| 2. Can you complete the core technical combinations of this activity smoothly and coherently? | ○ | ○ | ○ | ○ | ○ | ○ | ○ |
| 3. In real play or confrontation, can you successfully apply the skills you have learned? | ○ | ○ | ○ | ○ | ○ | ○ | ○ |

### **B. Perceived Competence**

| Item | 1 | 2 | 3 | 4 | 5 | 6 | 7 |
| --- | --- | --- | --- | --- | --- | --- | --- |
| 1. I think I am good at this sport/activity. | ○ | ○ | ○ | ○ | ○ | ○ | ○ |
| 2. In practicing this sport/activity, my performance is satisfactory. | ○ | ○ | ○ | ○ | ○ | ○ | ○ |
| 3. I feel confident about my ability in this sport/activity. | ○ | ○ | ○ | ○ | ○ | ○ | ○ |
| 4. Compared with others, I feel that learning this sport/activity is not difficult for me. | ○ | ○ | ○ | ○ | ○ | ○ | ○ |
| 5. Mastering the skills of this sport/activity is something I can achieve. | ○ | ○ | ○ | ○ | ○ | ○ | ○ |

### **C. Perceived Autonomy Support from the Teacher/Coach**

| Item | 1 | 2 | 3 | 4 | 5 | 6 | 7 |
| --- | --- | --- | --- | --- | --- | --- | --- |
| 1. The teacher/coach gave me opportunities to choose how to do things. | ○ | ○ | ○ | ○ | ○ | ○ | ○ |
| 2. I feel that the teacher/coach understands my thoughts. | ○ | ○ | ○ | ○ | ○ | ○ | ○ |
| 3. The teacher/coach encouraged me to ask questions. | ○ | ○ | ○ | ○ | ○ | ○ | ○ |
| 4. The teacher/coach listened carefully to my opinions. | ○ | ○ | ○ | ○ | ○ | ○ | ○ |
| 5. Before giving instructions, the teacher/coach explained the reasons for doing so. | ○ | ○ | ○ | ○ | ○ | ○ | ○ |

### **D. Situational Interest**

| Item | 1 | 2 | 3 | 4 | 5 | 6 | 7 |
| --- | --- | --- | --- | --- | --- | --- | --- |
| 1. The content of this class felt very novel to me. | ○ | ○ | ○ | ○ | ○ | ○ | ○ |
| 2. The learning activities in this class strongly captured my attention. | ○ | ○ | ○ | ○ | ○ | ○ | ○ |
| 3. In this class, I felt both excited and interested. | ○ | ○ | ○ | ○ | ○ | ○ | ○ |
| 4. This class made me want to learn more knowledge about this sport/activity. | ○ | ○ | ○ | ○ | ○ | ○ | ○ |
| 5. During practice, I felt time passed quickly. | ○ | ○ | ○ | ○ | ○ | ○ | ○ |

### **E. Behavioral Engagement & Intention to Improve Skills**

| Item | 1 | 2 | 3 | 4 | 5 | 6 | 7 |
| --- | --- | --- | --- | --- | --- | --- | --- |
| 1. In this class, I worked very hard to practice every movement. | ○ | ○ | ○ | ○ | ○ | ○ | ○ |
| 2. When I encounter a difficult movement, I persist until I can do it correctly. | ○ | ○ | ○ | ○ | ○ | ○ | ○ |
| 3. I actively think about how to improve my movement techniques. | ○ | ○ | ○ | ○ | ○ | ○ | ○ |
| 4. I plan to continue practicing this sport/activity in the coming period. | ○ | ○ | ○ | ○ | ○ | ○ | ○ |
| 5. Even without any mandatory requirement, I am willing to practice this sport/activity outside of class time. | ○ | ○ | ○ | ○ | ○ | ○ | ○ |

**End of the questionnaire.**
**Thank you very much for your participation and support!**
